# Training competencies in adult metabolic medicine: A survey of working adult metabolic medicine physicians

**DOI:** 10.1002/jmd2.12312

**Published:** 2022-06-30

**Authors:** Sandra Sirrs, Elisa Fabbro, Annalisa Sechi, for the SSIEM Adult Metabolic Medicine Training Competencies Working Group

**Affiliations:** ^1^ Division of Endocrinology, Department of Medicine University of British Columbia Vancouver British Columbia Canada; ^2^ Research and Third Mission Unit University of Trieste Trieste Italy; ^3^ Regional Coordinating Center for Rare Diseases University Hospital of Udine Udine Italy

**Keywords:** adult metabolic medicine, objectives of training, postgraduate medical education, training competencies

## Abstract

The rapid expansion of the number of adult patients with inherited metabolic diseases (IMDs) has created demand for physicians with expertise in the field of adult metabolic medicine (AMM). Unfortunately, existing accredited training programs in this field are rare, and training programs in pediatric metabolic medicine cannot fully meet the needs of AMM physicians as the types of patients and the problems they face are different in the adult setting. We surveyed a group of working practitioners in AMM for input on what medical expert competencies they feel should be included as part of training programs in AMM. Through a modified Delphi process, 66 physicians from six continents reached consensus on a comprehensive list of training competencies in AMM. This list includes competencies from the fields of adult internal medicine, neurology, medical genetics, and pediatric metabolic medicine but also includes competencies not found in any of those programs, leading to the conclusion that the training needs for specialists in AMM cannot be met from any of these existing programs. We propose that AMM be considered a subspecialty separate from pediatric metabolic medicine and that accredited training programs in AMM be created using these medical expert competencies as part of a broader program design.

**Synopsis**

We have developed a list of medical expert competencies for specialists in adult metabolic medicine using input from working clinicians in this field.

## 1 BACKGROUND

Adult metabolic medicine (AMM) is a relatively new discipline focusing on the care of adult patients with inherited metabolic diseases (IMDs). Clinical care of adults with IMDs has emerged as a new challenging reality.^1^ Data suggest that more than 50% of patients being followed worldwide are now adults.^2^ Despite this increasing demand, very few countries have dedicated training programs in the specialty of AMM^3^ and this is compromising the care of both those patients whose disorders are diagnosed as adults and those who are transitioned from the pediatric metabolic medicine clinics.^4^ The training available to most working AMM physicians is based within pediatric focused programs. However, as the types of patients seen by the adult clinics^5^ and the problems that develop in these patients as they age differ from those in children, this pediatric focussed training does not meet training needs. A recent survey of working AMM physicians showed that 73% of those surveyed felt that education they received prior to starting work was rated fair or poor and 95% felt that they were essentially “learning on the job.”^6^ We surveyed working AMM physicians to determine what they felt trainees in AMM should learn, using the experience of these physicians to develop a list of training competencies in AMM which then could be used to guide the establishment of formal training programs to meet the increasing demands for care for this patient population.

## 2 METHODS

We consolidated training competencies for Canadian programs in medical genetics,^7^ general internal medicine,^8^ pediatric biochemical genetics,^9^ and those available at the start of this process for the only currently available accredited training program in AMM in the United Kingdom.^10^ In August 2021 the UK objectives in AMM were updated^11^ but, as our survey was already completed, the updated version of the UK training competencies in AMM was not included in this survey. Canadian specialty training programs utilize the CanMEDS framework for medical education which includes multiple different roles (professional, communicator, collaborator, health advocate, scholar, leader) which overlap with the role of “medical expert.”^12^ We recognize that different countries adhere to different frameworks, but all of these will include a category equivalent to “medical expert.” We chose therefore to focus our survey on the medical expert category only, reasoning that training programs in different countries would need to develop separate competencies for the other roles which are in keeping with their own national frameworks for subspecialty medical education.

**FIGURE 1:**
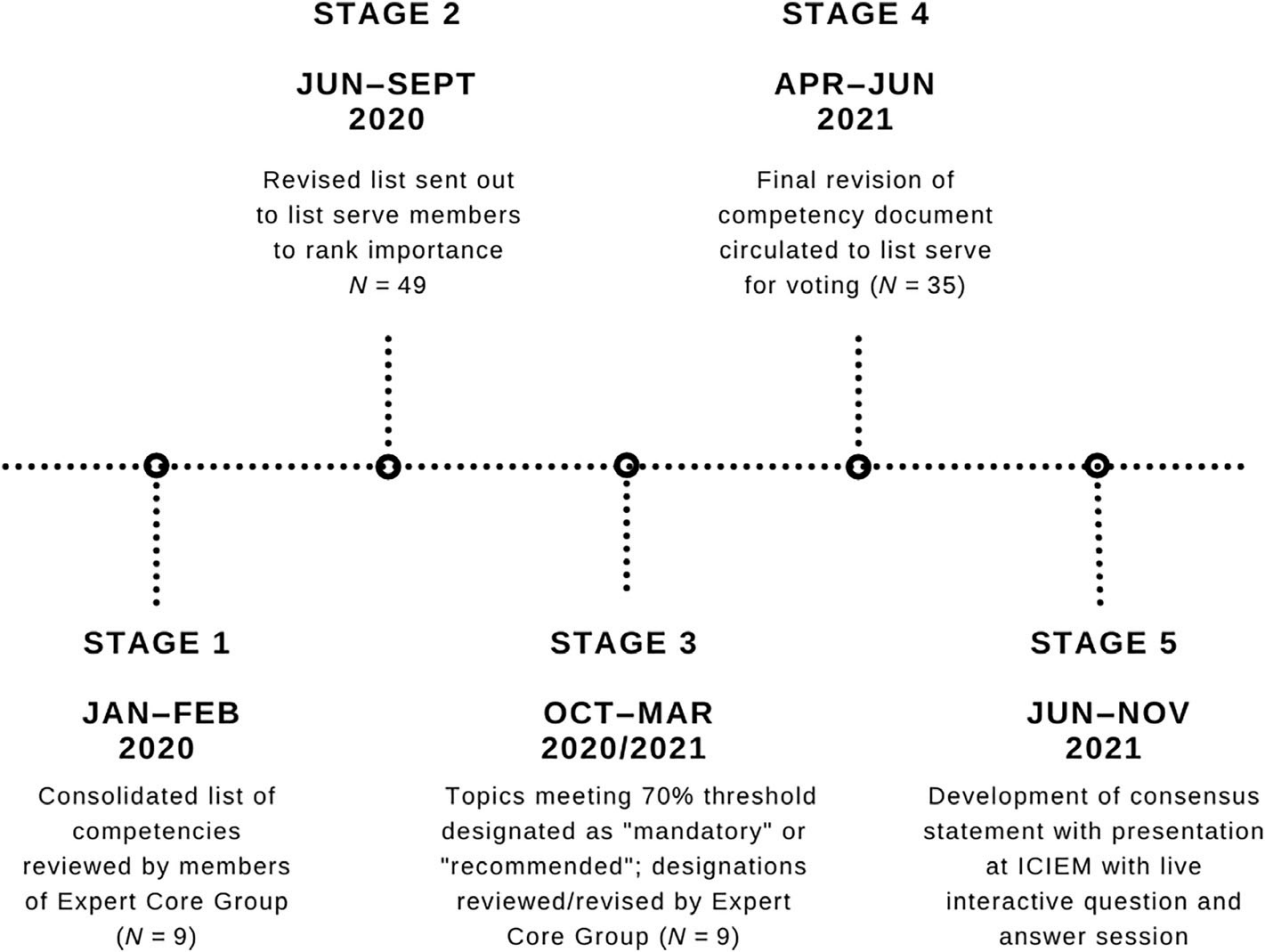
Stages in development of consensus statement for training competencies in adult metabolic medicine

The consolidated list of medical expert competencies then underwent a multistage process (Figure 1) to reach a consensus statement in a modified Delphi method.^13^, ^14^ This process took longer than originally anticipated due to delays imposed by the Covid‐19 pandemic.

### 2.1 Stage 1 (January–February 2020)

The list was reviewed by a core group of highly experienced AMM physicians (Faghfoury, Hollak, Lachmann, Langeveld, Lehman, Mochel, Murphy, Moc Tchan, Wilcox) from large AMM centers from five different countries. Reviewers were asked which competencies they felt were of little relevance and could be dropped from the list as well as for suggestions for medical expert topics they felt were not included in the consolidated list. These suggestions were incorporated into the first revision of the list (shown in the Appendix S1).

### 2.2 Stage 2 (June–September 2020)

An online questionnaire was developed and distributed by email to members of the AMM list serve which is an email group affiliated with the Society for the Study of Inborn Errors of Metabolism (SSIEM). Respondents were asked to rank the competencies from the first revised list as to their relevance using a 4‐point rating scale (not important, of little importance, important, very important), and were also able to add new training competencies if they felt these had been missed from the list.

### 2.3 Stage 3 (October 2020–March 2021)

Those topics rated as “important” or “very important” by fewer than 70% of the survey respondents in Stage 2 were dropped from the list. Those topics meeting the 70% threshold were then subdivided into competencies viewed as “mandatory” (ranked as “very important” by 50% or more of respondents) and “recommended” (ranked as “important” or “very important” by >70% but as “very important” by <50% of survey respondents). These designations were then reviewed again by the core group of experienced physicians who could recommend changes in the designations from “recommended” to “mandatory,” leading to the creation of the second revision of the competency document.

### 2.4 Stage 4 (April–June 2021)

The second revision of the competency document was circulated by email to the full AMM list serve and respondents were asked if they did or did not support this list of training competencies using an on‐line voting tool.

### 2.5 Stage 5 (June–October 2021)

A final consensus document with the list of mandatory and elective competencies for specialists in adult metabolic medicine was developed (see Appendix S1). The final document was then presented virtually at the International Congress of Inborn Errors of Metabolism (ICIEM, November 21–24, 2021) with a live interactive question and answer session as part of that presentation.

## 3 RESULTS

The consolidated list of competencies included competencies in 10 medical expert domains including basic science in cellular biology and genetics, consultative expertise in AMM, clinical expertise in IMDs in adults, appropriate use of laboratory testing, longitudinal care of adults with IMDs, treatment, management of contraception, pregnancy and lactation, transition management, management of complications, and critical appraisal. Forty‐nine working AMM physicians participated in Stage 2 and 35 physicians in Stage 4. Not all physicians participated in both Stages 2 and 4 so, in total, 66 working AMM physicians (55% of the 119 physicians who participate in the SSIEM email list group) participated in one or more of the phases of this project. Demographic details and clinical experience of the respondents in Stage 2 are included in Table S1. We received input from six continents although most respondents were from Europe and Canada. The most common specialties reported by respondents were internal medicine (*N* = 18), medical genetics (*N* = 14) and metabolic medicine (*N* = 10). Some respondents reported more than one area of specialty training. These respondents were highly experienced, with 60% having 10 or more years in practice and almost half of respondents were in settings which followed 500 or more patients.

There was widespread agreement among respondents with 95.2% of the 166 competencies identified in first revision ranked as important or very important by 70% or more of respondents. Table 1 shows the top competencies in each of the 10 domains and the full list of competencies meeting the 70% threshold can be seen in Appendix S1. Some of these competencies are common to both pediatric and adult IMD practice (e.g., hypoglycemia, hyperammonemia, lactic acidosis). However, others focus on clinical scenarios more familiar to adult physicians (stroke, chest pain, pregnancy, etc.) whereas scenarios important in the practice of pediatric metabolic medicine (such as sudden unexpected death) were ranked as less important for adult practice. As expected, there is overlap of the competencies prioritized by AMM physicians (e.g., knowledge of specific IMD disorders) with some of the knowledge‐based competencies in the UK AMM training curriculum^10^ and pediatric biochemical genetics.^9^ However, neither of those two programs identified other competencies prioritized by AMM physicians related to internal medicine and critical appraisal skills related to rare diseases.

**TABLE 1:** Top ranked competencies in each area

Area of competency	% of Respondents ranking as very important
Area 1: Basic Science in cellular biology and genetics	
*Describe and discuss the general concepts of human biochemistry and molecular biology, including*:	
Fluid and electrolyte balance, acid–base regulation, intermediary metabolism and metabolic response to fed and fasting states	70
Enzymes/proteins: structure/function relationships, cellular distribution, mechanisms of mechanisms of action, control of enzyme activity, role of cofactors, principles of measurement, enzyme kinetics	58
Demonstrate effective, appropriate, and timely performance of diagnostic procedures relevant to adult metabolic medicine, including but not limited to skin biopsy and lumbar puncture	57
Area 2: Consultative expertise in Adult Metabolic Medicine	
Carry out a comprehensive physical examination including both general and detailed neurological examination^a^	89
*Demonstrate the ability to assess and order appropriate investigations for common presentations including*:	
Hyperammonemia^a^	89
Lactic acidemia	81
Area 3: Clinical expertise in inherited metabolic diseases in adults	
*Demonstrate an understanding of the pathological and biochemical changes, clinical symptoms, investigations in metabolic disorders of the following pathways*:	
Hyperammonemia and urea cycle disorders^a^	89
Lysosomal storage disorders^a^	87
Disorders of carbohydrate metabolism: glycogen storage diseases, galactosemia, fructose intolerance	85
Disorders of fatty acid oxidation and carnitine metabolism	85
Area 4: Appropriate use of laboratory testing	
*Demonstrate proficiency in the appropriate indications for and limitations of testing, and demonstrate a familiarity and broad understanding of the methodologies as they relate to result interpretation of*:	
Intermediary metabolites (glucose, ammonia, lactate, pyruvate, free fatty acids, homocysteine, ketones)	85
Amino acids	83
Organic acids	83
Carnitine/acylcarnitines	83
Area 5: Longitudinal care of adults with inherited metabolic disorders	
Demonstrate knowledge of the appropriate indications for emergency /crisis management of metabolic disorders^a^	96
Identify appropriate investigations and timing for clinical, laboratory and imaging investigations for the long term follow up of adults with inherited metabolic diseases	83
Demonstrate an ability to work in multi‐disciplinary teams with biochemists, dieticians, etc.	83
Identify the risk of metabolic decompensation in patients who develop new medical problems either related or unrelated to their IMD (e.g., cancer, coronary artery disease, diabetes mellitus, etc.)	83
Area 6: Treatment^b^	
Understand available drug therapies for inherited metabolic disorders including the indications to initiate and discontinue the medications, mechanism of action, and cost^a^	91
Understand the principles of treatment related to inborn errors of metabolism^a^	89
Demonstrate an ability to start treatment to manage acute metabolic decompensation while diagnostic investigations are in process^a^	89
Understand acute and chronic side effects of the specific drug treatments and be able to manage them^a^	87
Area 7. Management of contraception, pregnancy and lactation	
Demonstrate the ability to optimize metabolic control in preconception for planned pregnancies (i.e., PKU)^a^	89
Demonstrate an ability to provide recommendations to pregnant adults with inherited metabolic diseases on diet, drug therapy, and appropriate investigations^a^	87
Understand the impact of common inherited metabolic diseases on fertility, maternal and fetal risks in pregnancy, and impact on lactation	81
Area 8: Transition management	
Understand the key issues in engaging young adults during the transition from pediatric to adult services	68
Understand developmental and behavioral barriers to adherence to treatment regimens in transitional aged youth and appropriate strategies to promote adherence	62
Outline the concept of patient self‐care and the role of the expert patient	47
Area 9: Management of complications	
Encephalopathy	74
Acid–base disturbances	72
Fluid and electrolyte abnormalities	68
Area 10: Critical appraisal^c^	
Apply skills in critical appraisal to the care of adult patients with inherited metabolic disorders	79
Understand the importance of critical appraisal in the evaluation of therapies for inherited metabolic disorders	77
Demonstrate the ability to manage uncertainty when faced with incomplete evidence to guide disease management	70

*Note*: The top three competencies in each of the 10 defined areas are shown. If there were more than three competencies with equivalent rank, all are shown.

^a^ Ten competencies receiving the highest ranking—note that the competencies of “hyperammonemia” and “hyperammonemia and urea cycle defects” overlap and are counted as one within the top 10.

^b^ Several competencies in nutritional therapy for inherited metabolic diseases (IMDs) were ranked as mandatory or recommended by survey respondents but were ranked below the top 3 so are not shown in this table but can be seen in Appendix S1.

^c^ All competencies under Area 10 were ranked as important or very important by more than 90% of survey respondents including those that are not shown in Table 1 but can be seen in the full list of competencies provided in the Appendix S1.

Table 1 shows the 10 training competencies which received the highest ranking of importance and, not surprisingly, these are focused on management of acute decompensation, clinical therapeutics, and pregnancy. There was also significant priority placed by respondents on competencies focused on skills of critical appraisal as they apply to rare diseases which were ranked as important or very important by >90% of respondents. We believe this reflects the high level of uncertainty faced by clinicians around therapies for rare diseases where tools used to assess treatments for common diseases (like large randomized placebo‐controlled trials) are often not available to aid in decision making.

In Stage 4, the competency document with revisions from Stage 3 was again circulated to the list‐serve and participants were asked to vote on the document using an on‐line voting tool. All 35 respondents in Stage 4 voted to approve the content of the document. Again, the respondents were from a very broad geographic distribution (Europe, United Kingdom, Canada, Australia, Qatar) and a wide variety of clinical backgrounds. The final approved competency document is included in Appendix S1.

## 4 DISCUSSION

We engaged working physicians in AMM to develop competencies for dedicated training in AMM. These competencies are available for use by any country involved in setting up accredited training in this field. The strengths of our project are the broad consultative input (66 working physicians from 6 continents) and the high level of clinical experience of our respondents who have real world experience in the field. Our project is limited though by focusing on medical expert competencies only so complete objectives of training which specify the other physician roles will need to be developed in individual countries. Also, our study has underrepresentation of some areas of the world, notably the United States. We did approach the Society for Inherited Metabolic Disorders (SIMD) which is a US based organization dedicated to the study of IMDs similar to the SSIEM. However, the SIMD does not have a specific adult subsection so we were unable to distribute our survey to clinicians who may be members of the SIMD but not the SSIEM.

Although not intended as a needs assessment for clinicians with expertise in AMM, it is notable that >40% of the respondents to Stage 2 of our survey were 50 years of age and over. This fact, when combined with the increased numbers of adult patients with IMDs who require expert care, underscores the need to rapidly expand the availability of accredited training programs in this area.

The competencies prioritized by our experienced clinicians are derived from multiple other disciplines. We can infer from this that none of the existing training programs (general internal medicine, medical genetics, pediatric biochemical genetics, or the UK program of AMM) are sufficient to provide the training thought necessary for consultants in AMM. Our responders put a high priority on training in critical appraisal of drugs for rare diseases. Such training would have to be a dedicated feature of training programs in AMM as the tools for appraisal of rare disease drugs differ from those for common drugs and therefore are not taught in internal medicine, general pediatrics, or medical genetics training programs. Such training would also be relevant to accredited training programs in pediatric metabolic medicine. Tools to facilitate training in this area (such as web‐based tutorials) could be developed by specialty organizations such as the SSIEM and made available to sites training in both pediatric and AMM.

Given the need for diverse training derived from multiple other specialties as well as specific training in areas relevant specifically to rare diseases which is not available in any other specialty, we propose that adult metabolic medicine should be recognized as a distinct subspecialty of medicine which can be reached through multiple specialty paths such as internal medicine or medical genetics. Subspecialty training programs in AMM require flexible curricula to accommodate the different specialty training backgrounds of residents. For example, all trainees would need to have training in the competencies specifically related to adult IMDs whereas trainees from medical genetics may need additional internal medicine training (neurology, intensive care, nephrology, etc.) and trainees from internal medicine would need medical genetics training (principles of genetics, variant interpretation, etc.). We suggest that any accredited training program in AMM should be located in a facility able to address the needs of trainees from different backgrounds. This specific subspecialty designation and flexible routes of entry are already available in the United Kingdom. The updated list of competencies specified by the existing UK AMM program^11^ includes more of those identified by our working physicians than the previous version.^10^ However, even the updated version has a relatively small amount of overlap with the competencies prioritized by our working AMM physicians so adjustment of the existing AMM training program would be required to address the competencies prioritized by our group of working AMM physicians (which included multiple members from the United Kingdom).

In conclusion, we have developed a consensus document on medical expert training competencies in AMM using a consultative process that drew on the experience of working physicians in this field. We hope these competencies can be used to stimulate the development of more accredited training programs in AMM.

## AUTHOR CONTRIBUTIONS

Sandra Sirrs, Elisa Fabbro, and Annalisa Sechi participated in all aspects of study design, protocol development, data analysis and manuscript development. Members of the SSIEM Adult Metabolic Medicine Training Competencies Working Group all participated in the validation of the training competencies and approved the final competency document which is included as supplementary material to the manuscript.

## FUNDING INFORMATION

No funding was received for this study.

## CONFLICT OF INTEREST

Sandra Sirrs, Elisa Fabbro, and Annalisa Sechi declare no conflicts of interest.

## ETHICS STATEMENT

No ethics approval was required for this study.

## INFORMED CONSENT

This article does not contain any studies with human or animal subjects performed by the any of the authors.

## Supporting information


**Appendix S1** First revision of the list at completion of stage 1

**Appendix S2** Adult Metabolic Medicine Competency document ‐ the approved version (Stage 4) including the full list of competencies voted on by survey participants

**Table S1** Characteristics of survey respondents in Stage 2.

## Data Availability

Data included in this manuscript are held by the authors.
