# Supplementary material for: Training competencies in adult metabolic medicine: A survey of working adult metabolic medicine physicians
Source: JIMD Rep. 2022 Jun 30;63(5):468–74. doi: 10.1002/jmd2.12312 (PMC9458608; doi:10.1002/jmd2.12312)
Supplement: Supplementary file 2 — Appendix S2 Adult Metabolic Medicine Competency document ‐ the approved version (Stage 4) including the full list of competencies voted on by survey participants [file JMD2-63-468-s003.docx]

**CONSENSUS DOCUMENT**

**Competencies for specialists in Adult Metabolic Medicine**

**Contributions and Acknowledgements**

Many individuals contributed to this list of competencies.

Firstly, we would like to acknowledge Sandra Sirrs (Canada), Elisa Fabbro (Italy) and Annalisa Sechi (Italy) who conceptualized this project, developed tools to facilitate input from members of the SSIEM Adult Metabolic Physicians Group, and collated these data into a draft guidelines document for consideration by the other contributors.

Secondly, we would like to thank the Society for the Study of Inborn Errors of Metabolism (SSIEM) for their support of the SSIEM Adult Metabolic Physicians email list group.

We would like to thank members of the expert core group - Hanna Faghfoury (Toronto, Canada), Carla Hollak (Amsterdam, the Netherlands), Robin Lachmann (London, United Kingdom), Mirjam Langeveld (Amsterdam, the Netherlands), Anna Lehman (Vancouver, Canada), Fanny Mochel (Paris, France), Elaine Murphy (London, United Kingdom), Michel Tchan (Sydney, Australia), Gisela Wilcox (Manchester, United Kingdom).

We would also like to thank participants from the SSIEM Adult Physicians Metabolic Medicine Training Competencies Working Group*: Aguiar P, Lisbon, Portugal; Ben-Omran T, Doha, Qatar; Bordugo A, Verona, Italy; Brodosi L, Bologna, Italy; Cassiman D, Leuven, Belgium; Chan AKJ, Edmonton, Canada; Chaves P, Sao Joao, Portugal; Dawson, C Birmingham, UK; De Jong Gerald, Melbourne, Australia; Douillard C, Lille, France; Durães J, Coimbra, Portugal; El Naga K, Medea, Algeria; Fazio T, Melbourne, Australia; Faghfoury H, Toronto, Canada; Galloway P, Glascow Scotland; Glamuzina E, Auckland, New Zealand; Gomes DCC, Lisbon, Portugal; Hollak CE, Amsterdam, the Netherlands; Hochuli M, Bern, Switzerland; Horvath G, Vancouver, Canada; Janssen MCH, Nijmegen, The Netherlands; Kantola I, Turku, Finland; Khan A, Calgary, Canada; Kwok M.K. Pok Fu Lam, Hong Kong, China; Lachmann R, London, United Kingdom; Langendonk J, Rotterdam, the Netherlands; Langeveld M, Amsterdam, the Netherlands; Lavigne C, Angers, France; Lehman A, Vancouver, Canada; Loughrey C, Belfast, Northern Ireland; Lourenco, C, Sao Paulo, Brazil; Maillot F, Tours, France; Marchi G, Verona, Italy; Marelli C, Montpellier, France; Mattman A, Vancouver, Canada; Meersseman W, Leuven, Belgium; Miller-Hodges E, Edinburgh, Scotland; Mochel F, Paris, France; Morel C, Toronto, Canada; Murphy E, London, United Kingdom; Nimmo G, Toronto, Canada; Oliveira JP, Porto, Portugal; Pastores G, Dublin, Ireland; Pintos-Morell G, Barcelona, Spain; Prasad C, London, Canada; Rahman S, London, United Kingdom; Rahman Y, Sydney, Australia, Reismann P, Budapest, Hungary; Ribeiro R, Porto, Portugal; Rockman-Greenberg C, Winnipeg, Canada; Sharma R, Salford, United Kingdom; Stepien KM, Salford, United Kingdom; Tarnopolsky M, Hamilton, Canada; Tchan M, Sydney, Australia; Vitturi N, Padova, Italy; Wagenmakers M, Rotterdam, the Netherlands; Wilcox G, Manchester, United Kingdom; Ziagaki A, Berlin, Germany

*Some survey participants preferred to remain anonymous and did not consent to the inclusion of their name in the working group

**Contents**

**Abbreviations Page 3**

**Background Page 4**

**Development process Page 4**

**Definitions of competency, mandatory competency, and recommended competency Page 5**

**Use and users Page 5**

**Table of competencies Page 6**

**Program structure Page 10**

**References Page 12**

**Abbreviations**

AMM Adult metabolic medicine

GIM General Internal medicine

IMD Inherited metabolic disease(s)

MedGen Medical Genetics

SSIEM Society for the Study of Inborn Errors of Metabolism

**Background**

Inherited metabolic diseases (IMD) are genetic disorders of intermediary metabolism. Although individual IMD are rare, collectively their birth prevalence is estimated to exceed 1/2000 and they are associated with substantial morbidity and mortality (1). Through improvements in diagnosis and therapy for genetic disorders, the number of patients with IMD who survive to transition to the adult health care system has increased markedly and up to 50% of IMD patients worldwide are now estimated to be adults (2). Adult metabolic medicine (AMM) is a relatively new specialty focusing on the care of adult patients with IMD. Despite the increasing demand, few countries have accredited training programs in the specialty of AMM (3) so most working AMM physicians receive training in pediatric focused programs. However, as the types of patients seen by the adult clinics (4) and the problems that develop in these patients as they age differ from those in children, this pediatric focused training does not meet all training needs. A recent survey of 42 working AMM physicians showed that 73% felt that education they received prior to starting work was rated poor or fair and 95% felt that they were essentially “learning on the job” (3). There is a need to define core competencies for specialists in AMM to facilitate the development of training programs to meet the clinical needs of the adult IMD population.

**Development process**

Objectives of training for Canadian training programs in medical genetics (MedGen [5]), general internal medicine (GIM [6])), pediatric biochemical genetics (7) and those available at the time of the survey for the only currently available accredited training program in AMM in the United Kingdom (8) were consolidated in a list of competencies. Although a recent update to training objectives for the UK AMM program was published in August 2021 (9), these were not available at the time of this process. Only those areas identified as “medical expert” in the CanMeds framework used by the Royal College of Physicians and surgeons in Canada (10) were considered.

The consolidated list of competencies then was reviewed by a core expert group of highly experienced AMM physicians from large AMM centers from 5 different countries. Reviewers in the core expert group were asked which competencies they felt were of little relevance and could be dropped from the list as well as for additional suggestions for medical expert topics which should be included. The revised list was then distributed as a survey to members of the AMM list serve (an email group affiliated with the Society for the Study of Inborn Errors of Metabolism [SSIEM]) where they were asked to rank the competencies as to their relevance using a 4-point rating scale (not important, of little importance, important, very important) and could make suggestions for topics that had been missed from the list.

Competencies that were ranked as important or very important by fewer than 70% of the members of the AMM list serve were then dropped from the list. Competencies that remained were then divided into two categories: “mandatory” competencies (those collectively ranked as important or very important by more than 70% of survey respondents and ranked as very important by 50% or more of survey respondents) and “recommended” competencies (those collectively ranked as important or very important by more than 70% of survey respondents but as very important by fewer than 50% of respondents). Feedback on the designation of individual competencies as “mandatory” or “recommended” was obtained from the core expert group through email, as face-to-face input was not possible due to pandemic restrictions. The designation of “mandatory” or “recommended” could be adjusted based on feedback from the core expert group. The final list of competencies was then sent to members of the AMM list serve who were asked to vote to accept or reject the list. The entire process took place between January 2020 and September 2021.

**Definitions of competency, mandatory competency, and recommended competency**

In this document, the term competency is defined as: the proven ability to use knowledge, skills and personal, social and/or methodological abilities, in work or study situations and in professional and personal development (11).

Given the high priority placed by survey respondents on competencies classified as “mandatory”, we propose that “mandatory competencies” should be considered as minimum pre-requisites for physicians seeking accreditation in the field of AMM and should be required as part of all accredited training programs.

While the thresholds are slightly lower for those competencies designated as “recommended”, the survey respondents feel that these competencies are nonetheless highly desirable for physicians specializing in AMM but acknowledge that limitations in training time requirements may preclude obtaining all of these competencies at the time of certification. Due to the rarity of these disorders, programs at different sites may need to collaborate to ensure adequate exposure for trainees. Competencies deemed as “recommended” which are not obtained at the time of certification can be used to guide continuing education of working consultants in AMM.

**Use and Users**

Although this document was developed primarily as an aide for the design of training programs in AMM**,** the list of competencies is a reference document that can be used for several purposes:

- Design and implementation of training programs in AMM within the boundaries of different national contexts
- Standardization of competencies for accreditation bodies which are evaluating proposals for specialty training in AMM
- Self-assessment of performance for clinicians working in the field of AMM
- Performance evaluation for clinicians working in the field of AMM
- Identification of the professional staff needs for health care organizations tasked with caring for adult patients with IMD

**Table 1. List of medical expert competencies in Adult Metabolic Medicine**

| **Area 1: Basic Science in cellular biology and genetics** |
| --- |
| **Mandatory** |
| ***Area 1.1: Describe and discuss the general concepts of human biochemistry and molecular biology, including:*** |
| 1.1.1 - Enzymes/proteins: structure/function relationships, cellular distribution, mechanisms of mechanisms of action, control of enzyme activity, role of cofactors, principles of measurement, enzyme kinetics |
| 1.1.3 - Structure/function relationships of intracellular components: nucleus, Golgi, endoplasmic reticulum |
| 1.1.4 - Regulation of intermediary metabolism including biochemical and hormonal regulation, and tissue compartmentalization |
| *Describe and discuss the general concepts of human physiology and biochemistry including:* |
| 1.1.5 - Fluid and electrolyte balance, acid-base regulation, intermediary metabolism and metabolic response to fed and fasting states |
| ***Area 1.2: Apply concepts of human heredity to clinical situations*** |
| **Mandatory** |
| 1.2.1 - Demonstrate effective, appropriate, and timely performance of diagnostic procedures relevant to adult metabolic medicine, including but not limited to skin biopsy and lumbar puncture |
| **Recommended** |
| 1.2.2 - DNA structure and function |
| 1.2.3 – Mechanisms of gene regulation expression |
| 1.2.4 - Variable impact of variants on protein function (e.g., hypomorphic or gain of function variants, dominant negative, haploinsufficiency etc.) |
| 1.2.5 - Perform pedigree analysis and risk calculation |
| ***Area 1.3: Understand available tools for genetic diagnosis including indications, limitations, and interpretation*** |
| **Mandatory** |
| 1.3.1 - Microarray, single gene testing (PCR and capillary sequencing) next generation technologies, WES/WGS etc |
| 1.3.2 - Variant types, interpretation and classification |
|  |
|  |
| **Area 2: Consultative expertise in Adult Metabolic Medicine** |
| **Mandatory** |
| *Perform a complete evaluation of patients presenting with disorders for which inherited metabolic diseases are in the differential diagnosis. In order to do this, the adult metabolic physician must be able to:* |
| 2.1 - Carry out a comprehensive physical examination including both general and detailed neurological examination |
| 2.2 - Identify nongenetic causes that could be contributing to the presentation |
| 2.3 - Identify dysmorphic features that may point to a diagnosis of an inherited metabolic disease |
| *Demonstrate the ability to assess and order appropriate investigations for common presentations including:* |
| 2.4 - Seizures |
| 2.5 - Developmental disability/ Premature onset Dementia/Cognitive decline |
| 2.6 - Acute and chronic encephalopathies |
| 2.7 - Movement disorders (ataxia, dystonia etc) |
| 2.8 - Abnormal brain imaging suggestive of a metabolic disorder (e.g., white matter disease, basal ganglia calcification etc.) |
| 2.9 - Psychiatric manifestations of metabolic disorders |
| 2.10 - Liver disease including acute liver failure, intrahepatic cholestasis, steatosis, cirrhosis, liver masses |
| 2.11 - Organomegaly |
| 2.12 - Cardiomyopathy |
| 2.13 - Eye disease, including corneal clouding, cataract, retinal changes, optic neuropathy, ophthalmoplegia |
| 2.14 - Muscle disease: myopathy, exercise induced rhabdomyolysis, exercise intolerance |
| 2.15 - Hypoglycemia |
| 2.16 - Metabolic acidosis |
| 2.17 - Hyperammonemia |
| 2.18 - Lactic acidemia |
| 2.19 - Premature stroke |
| **Recommended** |
| 2.20 - Neuropathies |
| 2.21 - Skeletal abnormalities of metabolic disorders (e.g., dysostosis multiplex, rhizomatic chondrodysplasia punctata, frequent fractures, osteoporosis) |
| 2.22 - Hair and skin manifestations of metabolic disorders (e.g., ichthyosis, angiokeratoma, lipomatosis) |
| 2.23 - Malodor (e.g., trimethylaminuria) |
| 2.24 - Renal disorders: Fanconi syndrome, recurrent renal calculi, proteinuria, renal failure |
| 2.25 - Sudden Unexpected death |
| **Area 3: Clinical expertise in inherited metabolic diseases in adults** |
| **Mandatory** |
| *Perform a complete evaluation of physiological and pathological states relevant to inborn errors of metabolism. In order to do this the adult metabolic physician must be able to:* |
| 3.1 - Appreciate clinical variation or phenotypic spectrum within genetic disorders |
| *Demonstrate an understanding of the pathological and biochemical changes, clinical symptoms, investigations in metabolic disorders of the following pathways:* |
| 3.2 - Disorders of amino acid metabolism |
| 3.3 - Disorders of organic acid metabolism |
| 3.4 - Hyperammonemia and urea cycle disorders |
| 3.5 - Disorders of carbohydrate metabolism: glycogen storage diseases, galactosemia, fructose intolerance |
| 3.6 - Disorders of fatty acid oxidation and carnitine metabolism |
| 3.7 - Disorders of ketone body metabolism |
| 3.8 - Lysosomal storage disorders |
| 3.9 - Peroxisomal disorders |
| 3.10 - Mitochondrial Respiratory chain disorders and disorders of pyruvate metabolism |
| 3.11 - Porphyrias |
| 3.12 - Disorders of cholesterol, sterol and bile acid metabolism |
| 3.13 - Disorders of vitamin metabolism (e.g., cobalamin) |
| 3.14 - Disorders of purine and pyrimidine metabolism  3.15 - Disorders of lipoprotein metabolism  3.16 - Disorders of metal metabolism  3.17 - Disorders of creatine metabolism  3.18 - Defects of membrane transport: cystinuria, lysinuric protein intolerance, etc.  3.19 - Disorders of glycosylation  3.20 - Disorders of neurotransmitters |
| **Recommended** |
| 3.21 - Metabolic bone diseases |
| **Area 4: Appropriate use of laboratory testing** |
| **Mandatory** |
| *Demonstrate proficiency in the appropriate indications for and limitations of testing, and demonstrate a familiarity and broad understanding of the methodologies as they relate to result interpretation of:* |
| 4.1 - Amino acids |
| 4.2 - Organic acids |
| 4.3 - Intermediary metabolites (glucose, ammonia, lactate, pyruvate, free fatty acids, homocysteine, ketones) |
| 4.4 - Carnitine/acylcarnitines |
| 4.5 - Enzymes studies: specific and non-specific assays (flux studies), respiratory chain enzymes |
| 4.6 - Glycosaminoglycans and oligosaccharides |
| 4.7 - Peroxisomal metabolites (VLCFA, phytanic, plasmalogens, bile acids, pipecolic acid) |
| 4.8 - Biomarkers (i.e., LysoGb3, oxysterols, chitotriosidase) |
| 4.9 - Mutation analysis, including mtDNA mutation analysis |
| 4.10 - Advise on indications, patient preparation, and result interpretation of loading, fasting, and other challenge tests for disorders of intermediary metabolism |
| 4.11 - Recommend appropriate enzymatic, biochemical or molecular carrier tests for metabolic disorders given the clinical context (family based, population based) |
| 4.12 - Demonstrate an ability to counsel patients and families on the risks and interpretation of genetic testing |
| 4.13 - Understand the impact of inherited metabolic disorders on common laboratory tests |
| 4.14 - Advise on the influence of clinical context and pre-analytical variables on test results: fasting, nutritional status, medications, age, gender, pregnancy, sampling procedure, etc |
| 4.15 - Transferrin isoforms, glycans and glycoprotein |
| 4.16 - Neurotransmitter and biogenic amine metabolites in CSF and other fluids |
| 4.17 - Purines and pyrimidines |
| **Area 5: Longitudinal care of adults with inherited metabolic disorders** |
| **Mandatory** |
| 5.1 - Identify appropriate investigations and timing for clinical, laboratory and imaging investigations for the long term follow up of adults with inherited metabolic diseases |
| 5.2 - Demonstrate knowledge of the appropriate indications for emergency /crisis management of metabolic disorders |
| 5.3 - Demonstrate an ability to work in multi-disciplinary teams with biochemists, dieticians etc |
| 5.4 - Provide continuity in care when indicated, and periodically assess the appropriateness of the care plan |
| 5.5 - Identify the risk of metabolic decompensation in patients who develop new medical problems either related or unrelated to their IMD (e.g., cancer, coronary artery disease, diabetes mellitus etc) |
| 5.6 - Demonstrate ability to collaborate with appropriate specialty colleagues to coordinate care of the IMD in the setting of the unrelated medical problem |
| 5.7 - Understand appropriate use of advanced care directions and palliative care services |
| 5.8 - Understand the psychological and psychosocial effects of IMDs and how they differ at different life stages |
| **Area 6: Treatment** |
| ***Area 6.1. Drug therapy*** |
| **Mandatory** |
| 6.1.1 - Understand the principles of treatment related to inborn errors of metabolism |
| 6.1.2 - Understand available drug therapies for inherited metabolic disorders including the indications to initiate and discontinue the medications, mechanism of action, and cost |
| 6.1.3 - Understand acute and chronic side effects of the specific drug treatments and be able to manage them. |
| *Demonstrate knowledge of the underlying principles, and skill in the application, of various dietary or pharmacological treatment strategies employed for metabolic disorders:* |
| 6.1.4 - Substrate reduction therapy |
| 6.1.5 - Correcting co-factor or product deficiency |
| 6.1.6 - Providing alternative substrates/promoting alternative pathways |
| 6.1.7 - Blocking effects of toxic metabolites |
| 6.1.8 - Stimulating residual enzyme activity |
| 6.1.9 - Enzyme replacement therapy |
| 6.1.10 - Organ and stem cells transplantation |
| 6.1.11 - Gene therapy |
| 6.1.13 - Demonstrate an ability to start treatment to manage acute metabolic decompensation while diagnostic investigations are in process |
| 6.1.14 - Counsel affected families on prevention and treatment of exacerbations |
| 6.1.15 - Oligonucleotides therapy |
| ***Area 6.2: Nutritional therapy and fluid management*** |
| **Mandatory** |
| 6.2.1 - Demonstrate the ability to choose the appropriate route for nutrition support |
| 6.2.2 - Prescribe appropriate enteral and intravenous feeding regimens and adjust as necessary as the patient’s clinical condition alters. |
| 6.2.3 - Monitor nutrition support patients to avoid refeeding syndrome and other biochemical complications |
| **Area 7. Management of contraception, pregnancy and lactation** |
| **Mandatory** |
| 7.1 - Understand the impact of common inherited metabolic diseases on fertility, maternal and fetal risks in pregnancy, and impact on lactation |
| 7.2 - Demonstrate an ability to provide recommendations to pregnant adults with inherited metabolic diseases on diet, drug therapy, and appropriate investigations |
| 7.3 - Understand the impact of pregnancy induced change in blood volume, hemodynamics, and cardio-respiratory and renal physiology on common inherited metabolic diseases |
| 7.4 - Understand indications for involvement of other specialists in the care of a pregnant adult with inherited metabolic diseases |
| 7.5 - Demonstrate the ability to optimize metabolic control in preconception for planned pregnancies (i.e. PKU) |
| 7.6 - Counsel adults with inherited metabolic disorders who desire to avoid pregnancy on contraceptive options appropriate for their condition |
| 7.7 - Counsel adults with inherited metabolic disorders on the impact of their condition on complications of pregnancy, risks to the fetus, and implications for lactation |
| **Area 8: Transition management** |
| **Mandatory** |
| 8.1 - Understand the key issues in engaging young adults during the transition from pediatric to adult services |
| 8.2 - Understand developmental and behavioral barriers to adherence to treatment regimens in transitional aged youth and appropriate strategies to promote adherence |
| 8.3 - Outline the concept of patient self-care and the role of the expert patient |
| **Recommended** |
| 8.4 - Understand the role of patient organizations in the care of patients with rare diseases |
| **Area 9: Management of complications** |
| **Mandatory** |
| 9.1 - Cardiomyopathies |
| 9.2 - Encephalopathy |
| 9.3 - Acid-base disturbances |
| 9.4 - Fluid and electrolyte abnormalities |
| 9.5 - Altered mental status and disorders of consciousness |
| **Recommended** |
| *Apply knowledge of the following systems, clinical scenarios, conditions, diseases, and therapies applicable to the care of patients with inherited metabolic disorders, including the manifestations, initial investigations, and management and appropriate involvement of external specialists:* |
| 9.6 - Chest pain |
| 9.7 - Palpitations |
| 9.8 - Syncope |
| 9.9 - Abnormal cardiac enzymes |
| 9.10 - Cardiac murmurs |
| 9.11 - Acute coronary syndromes and their complications |
| 9.12 - Congestive heart failure |
| 9.13 - Pulmonary hypertension |
| 9.14 - Valvular heart disease |
| 9.15 - Hypertension |
| 9.16 - Acute and chronic dyspnea |
| 9.17 - Interstitial lung disease |
| 9.18 - Acute and chronic abdominal pain |
| 9.19 - Dysphagia |
| 9.20 - Nausea and vomiting |
| 9.21 - Ascites |
| 9.22 - Jaundice |
| 9.23 - Abnormal liver tests |
| 9.24 - Acute and chronic diarrhea |
| 9.25 - Hepatic neoplasms |
| 9.26 - Proteinuria |
| 9.27 - Acute renal failure |
| 9.28 - Chronic kidney disease and its complications |
| 9.29 - Nephritic and nephrotic syndromes |
| 9.30 - Renal calculi |
| 9.31 - Renal tubular acidosis |
| 9.32 - Male hypogonadism |
| 9.33 - Acute and chronic headache |
| 9.34 - Dizziness and vertigo |
| 9.35 - Syncope |
| 9.36 - Tremors |
| 9.37 - Cerebral vascular disease: stroke and transient ischemic attack (TIA) |
| 9.38 - Peripheral neuropathy |
| 9.39 - Anemia |
| 9.40 - Pancytopenia |
| 9.41 - Gout and pseudogout |
| 9.42 - Severe drug reactions including but not limited to anaphylaxis and toxic epidermal necrolysis |
| **Area 10: Critical appraisal** |
| **Mandatory** |
| 10.1 - Understand the importance of critical appraisal in the evaluation of therapies for inherited metabolic disorders |
| 10.2 - Demonstrate an understanding of limitations of conventional clinical trial design in the evaluation of rare diseases |
| 10.3 - Describe the advantages and disadvantages of alternate methods of clinical trial design in the evaluation of therapies for rare diseases |
| 10.4 - Demonstrate the ability to manage uncertainty when faced with incomplete evidence to guide disease management |
| 10.5 - Apply skills in critical appraisal to the care of adult patients with inherited metabolic disorders |
| **Recommended** |
| 10.6 - Understand the use and limitations of common tools used in evidence-based drug evaluation (ICER, QALY etc) |

**Program Structure**

Physician trainees in AMM come from a variety of backgrounds, most commonly from internal medicine (or its subspecialties) and medical genetics. Training programs in AMM will need flexibility in program structure based on the training background of physician trainees. The exposure to various acute and chronic medical disorders as part of general training in medical genetics and/or internal medicine will also vary by country. Regional training experiences will need to be considered when structuring a training program in AMM to ensure that the mandatory competencies can be achieved by trainees. In some centers, exposure to some areas of AMM may be restricted by factors including population size and such centers are encouraged to collaborate with other accredited training programs in larger centers to ensure adequate exposure of physician trainees. Finally, some trainees from programs such as medical genetics or internal medicine may have chosen elective experiences in their subspecialty training program that are relevant to training in AMM and the past training experiences of individual trainees can be considered in individualizing training programs so that all applicants can meet these competencies.

Although the actual structure of training programs in AMM will vary by country and by site, one possible model for a subspecialty training program in AMM which can incorporate trainees from different backgrounds is shown below as an example. The length of time trainees spend on any given rotation will vary according to their previous experience and training requirements in their country. For example, some internal medicine trainees may have already had exposure to high risk pregnancy programs or medical genetics trainees may have done rotations in nephrology etc. The initial part of the training program is intended to bring trainees from different backgrounds to a minimum common level and should be adapted to each trainee based on their past experience.

**Table 2. Sample program for subspecialty training in AMM to accommodate trainees from different specialty backgrounds**

| **Experiences for trainees from different backgrounds (first part of program)** | |
| --- | --- |
| **Trainees from an internal medicine background** | **Trainees from a Medical Genetics background** |
| Training in medical genetics which will include exposure to variant interpretation, pedigree analysis, general concepts of human heredity, prenatal counseling, and experience in an accredited molecular genetics laboratory; training in high risk pregnancy programs | Training in internal medicine which may include rotations in ICU, nephrology, endocrinology, cardiology, neurology and general internal medicine teaching units |
| **Experiences for trainees from all backgrounds** (second part of program)** | |
| Longitudinal clinic in AMM* | |
| Pediatric metabolic medicine to include both inpatient and outpatient exposure | |
| Biochemical genetics laboratory | |
| General clinics in AMM to include exposure to patients with confirmed diagnoses and those under investigation as well as experience with all nutritional aspects involved in the care of patients with metabolic disorders who receive medical nutrition therapy | |

*Although trainees from different backgrounds may initially largely be on other services, a ½ day longitudinal clinic in AMM running for the entire training period would be considered highly desirable.

**Additional rotations in gastroenterology/hepatology/liver transplantation and clinical nutrition focusing on enteral and parenteral forms of nutrition support would be considered as highly desirable.

**References**

1. Waters D. Adeloye D. Woolham D. Wastnedge E. Patel S and Rudan I. Global birth prevalence and mortality from inborn errors of metabolism: a systematic analysis of the evidence. J Glob Health. 2018;8:021102.
2. SCIENTIFIC REPORT. Board Annual Meeting. MetabERN. April 9th - 10th. 2018. Frankfurt - GERMANY
3. Sechi A. Fabbro E. Langeveld M. Tullio A. Lachmann R. Mochel F and the SSIEM Adult Physician Metabolic Group. Education and training in adult metabolic medicine: Results of an international survey. JIMD Reports 2019. DOI: 10.1002/jmd2.12044
4. Sirrs. S. Hollak C. Merkel M. Sechi A. Glamuzina E. Janssen MC. Lachmann R. Langendonk J. Scarpelli M. Ben Omran T. Mochele F. Tchan MC. The frequencies of different inborn errors of metabolism in adult metabolic centres: Report from the SSIEM Adult Metabolic Physicians Group. JIMD Reports. 2015;27: 85-91
5. Royal College of Physicians and Surgeons of Canada. Objectives of training in the specialty of medical genetics and Genomics version 1.2 May 2016. <http://www.royalcollege.ca/rcsite/documents/ibd/medical-genetics-genomics-otr-e> (downloaded Dec 13 2019).
6. Royal College of Physicians and Surgeons of Canada. Internal medicine competencies version 1.0 2018. <http://www.royalcollege.ca/rcsite/documents/ibd/medical-genetics-genomics-otr-e> (downloaded Dec 13 2019).
7. Canadian College of Medical Geneticists. CCMG Clinical biochemical genetics training guidelines and specialty requirements July 1 2014. <https://www.jrcptb.org.uk/specialties/metabolic-medicine-sub-specialty> (downloaded Dec 13 2019).
8. Joint Royal Colleges of Physicians Training Board. Training curriculum for the sub-specialty of metabolic medicine August 2010. <https://www.jrcptb.org.uk/specialties/metabolic-medicine-sub-specialty> (downloaded Dec 13 2019).
9. Royal College of Pathologists United Kingdom. Curriculum for specialty training in chemical pathology: Appendix A. 2021:  <https://www.gmc-uk.org/education/standards-guidance-and-curricula/curricula/chemical-pathology-curriculum> (downloaded Nov 24 2021).
10. Royal College of Physicians and Surgeons of Canada. CanMEDS Framework 2015. <http://www.royalcollege.ca/rcsite/canmeds/canmeds-framework-e> (downloaded Oct 5 2010).
11. European Center for Disease Prevention and Control. Core competencies for infection control and hospital hygiene professionals in the European Union. Stockholm. ECDC. 2013. Doi 10.2900/7778 (downloaded Dec. 13 2019).
