# Supplementary material for: Training competencies in adult metabolic medicine: A survey of working adult metabolic medicine physicians
Source: JIMD Rep. 2022 Jun 30;63(5):468–74. doi: 10.1002/jmd2.12312 (PMC9458608; doi:10.1002/jmd2.12312)
Supplement: Supplementary file 3 — Table S1 Characteristics of survey respondents in Stage 2. [file JMD2-63-468-s002.docx]

| Characteristic | Number of respondents (%) |
| --- | --- |
| Age group (years) | |
| <30 | 1 (2%) |
| 30-39 | 9 (18%) |
| 40-49 | 16 (33%) |
| 50-59 | 14 (29%) |
| 60 -69 | 4 (8%) |
| 70 or greater | 1 (2%) |
| Missing information | 4 (8%) |
| Years in practice of AMM | |
| 1-5 | 8 (16%) |
| 6-10 | 11 (22%) |
| >10 | 28 (57%) |
| Missing information | 2 (4%) |
| Number of adult IMD patients followed | |
| <100 | 1 (2%) |
| 101-250 | 7 (14%) |
| 251-500 | 17 (35%) |
| 501-1000 | 13 (27%) |
| 1001-2000 | 3 (6%) |
| >2000 | 6 (12%) |
| Missing information | 2 (4%) |
| Speciality training background^1^ | |
| Internal medicine | 18 |
| Medical genetics | 14 |
| Metabolic medicine (age unspecified) | 10 |
| Pediatrics | 9 |
| Endocrinology | 7 |
| Neurology | 4 |
| Chemical pathology | 4 |
| Nephrology | 2 |
| Pediatric genetics and metabolic medicine | 2 |
| Vascular medicine^2^ | 1 |
| Nutrition | 1 |
| Missing information | 2 |

**Supplementary Table 1. Characteristics of survey respondents in Stage 2**

^1^Note that some survey respondents identified more than one area of specialty training so percentages are not given. Participants self-identified their training so the terms used to describe specialty training backgrounds may not be common in all countries and some terms may overlap.

^2^In the United Kingdom, the specialty of “General and Vascular Medicine” is a nonsurgical discipline included in the list of other subspecialties more commonly considered to be part of “internal medicine” – for details, see https://www.rcpe.ac.uk/careers-training/sas-general-and-vascular-medicine
